# Dissection and internal anatomy of the giant tropical bont tick *Amblyomma variegatum*

**DOI:** 10.1051/parasite/2025068

**Published:** 2025-11-28

**Authors:** Naomie Pature, Nonito Pagès, Valérie Rodrigues, Damien F. Meyer

**Affiliations:** 1 CIRAD, UMR ASTRE F-97170 Petit-Bourg Guadeloupe France; 2 ASTRE, CIRAD, INRAE, Université de Montpellier F-34398 Montpellier France; 3 Université des Antilles, École doctorale n° 636 DEECA, Campus de Fouillole F-97157 Pointe-à-Pitre Guadeloupe France; 4 CaribVET, site de Duclos 97170 Petit-Bourg Guadeloupe France

**Keywords:** *Amblyomma variegatum*, Heartwater, Dissection, Tick organs, Anatomy, Hard-bodied ticks

## Abstract

The tropical bont tick, *Amblyomma variegatum*, is endemic in sub-Saharan and southern Africa, as well as several Caribbean islands. This tick is deleterious for the general health of ruminants and is the primary vector of *Ehrlichia ruminantium*, the causative agent of heartwater. Understanding the ecology and biology of *A. variegatum* is thus crucial to describe tick-host-bacteria interactions and thus develop effective control methods. To better understand vector competence, detailed functional characterization and precise isolation of tick organs is needed. All procedures described in this study were performed using a simple, high-quality binocular magnifying glass. Following this approach, we present descriptions and illustrations of the internal anatomy of *A. variegatum* at male, female (at various stages of engorgement), and nymph stages. This study focused on critical tissues associated with vector competence, including midguts, salivary glands, or ovaries, which were targeted and isolated. We identified morphological differences in the reproductive systems of both *A. variegatum* male and female ticks when compared with other tick species. We also provide numerous practical and technical aspects for obtaining organs suitable for detailed studies. The method presented here ensures organs of high quality, without degradation or contamination, as required for cellular or molecular studies on host-vector-pathogen interactions.

## Abstract (fr)

La tique tropicale *Amblyomma variegatum* est endémique en Afrique subsaharienne et australe ainsi que dans plusieurs îles des Caraïbes. Elle est délétère pour la santé générale des ruminants et constitue le principal vecteur d*’Ehrlichia ruminantium*, l’agent causal de la cowdriose. Il est donc essentiel de comprendre l’écologie et la biologie d’*A. variegatum* afin de décrire les interactions entre la tique, l’hôte et la bactérie et de mettre au point des méthodes de lutte efficaces. Pour mieux comprendre la compétence vectorielle, il est nécessaire de procéder à une caractérisation fonctionnelle détaillée et à un isolement précis des organes de la tique. Toutes les procédures décrites dans cette étude ont été réalisées à l’aide d’une simple loupe binoculaire de haute qualité. Nous présentons ainsi des descriptions et des illustrations de l’anatomie interne des stades mâle, femelle (à différents stades de gorgement) et nymphe d’*A. variegatum*. Cette étude s’est concentrée sur les tissus clés associés à la compétence vectorielle, incluant l’intestin moyen, les glandes salivaires ou les ovaires, qui ont été ciblés et isolés. Nous avons identifié des différences morphologiques au niveau des systèmes reproducteurs des tiques mâles et femelles *A. variegatum* par rapport à d’autres espèces de tiques. Nous expliquons également de nombreux aspects pratiques et techniques pour obtenir des organes adaptés à des études détaillées. La méthode présentée ici garantit des organes de haute qualité, sans dégradation ni contamination, comme l’exigent les études cellulaires ou moléculaires pour les interactions hôte-vecteur-pathogène.

## Introduction

The tropical bont tick, *Amblyomma variegatum* Fabricius, 1794 is an obligate hematophagous arthropod belonging to the phylum Arthropoda, subphylum Chelicerata, Class Arachnida, subclass Acari, and order Ixodida [16]. This tropical tick is also referred to as the Senegalese tick in the Guadeloupe archipelago and the Antigua gold tick in Antigua [50]. *Amblyomma variegatum* belongs to the family Ixodidae, characterized by a hard body with a dorsal shield (scutum) that may be ornamented in males, or not ornamented in females.

*Amblyomma variegatum* acts as the primary vector for heartwater, a major and often fatal disease of ruminants in sub-Saharan and southern Africa, and in the Caribbean. This important disease is caused by the bacterium *Ehrlichia ruminantium*. The presence of this tick also contributes to lameness and the formation of deep abscesses in small ruminants, often leading to immunosuppression and metabolic disorders [9] and is frequently associated with dermatophilosis [51]. These impacts result in considerable economic and health burdens. While more than ten species of *Amblyomma* ticks transmit *E. ruminantium* in Africa, *A. variegatum* and *A. hebraeum* are the main vectors [10]. The geographic overlap of heartwater-endemic regions with *Amblyomma* distribution underscores the importance of studying these ticks. Factors such as their abundance, activity, and environmental adaptability directly influence their vector potential [69]. Beyond endemic areas, *A. variegatum* also poses a potential threat to non-endemic regions due to livestock movement and climate change [24].

Knowing the tick’s anatomy is therefore critical for studying tick-pathogen interactions and developing appropriate vector control strategies to mitigate the socioeconomic and health impacts of tick-borne diseases, which disproportionately affect resource-limited regions. While research on *Rhipicephalus microplus* and *Ixodes scapularis* have provided valuable insights into tick physiology, *A. variegatum* remain relatively understudied. Given the widespread distribution of *A. variegatum*, particularly on some Caribbean islands, there is a need to study the tick and its role as a pathogen vector.

The study of the internal anatomy of arthropods is of great interest due to the remarkable diversity observed, particularly regarding the specific barriers such as target organs they possess. The precise identification of tick internal organs is also of significant value in entomology and microbiology. Despite the availability of numerous dissection guides for identifying tick organs, especially for *Rhipicephalus* species, comparatively few have focused on *Amblyomma* ticks [18, 68]. High-quality histologic images of the reproductive and digestive systems of ixodid ticks are readily available [16, 39, 70], along with scanning electron microscopy images of male and female *Amblyomma* reproductive structures [6, 17]. However, beyond the seminal work by Edwards [22], dissection-based studies on *Amblyomma* ticks remain scarce. Even the pictorial instructions provided in this guide rely on two different *Amblyomma* species to provide a comprehensive view of *Amblyomma* internal organs.

In this study, the internal structures of nymph and adult *A. variegatum* ticks are described and illustrated, at different stages of engorgement. Further anatomical comparisons between several species of ticks, like *Amblyomma* ticks, in the literature, are provided to highlight the uniqueness of *A. variegatum* ticks. This approach was selected to ensure a reliable comparison between tick species within the same genus, which are susceptible to share more common features.

## Material and Methods

### Dissection tools

The materials necessary for tick dissections are detailed in the “Tick general dissection” section below. All the dissection tools were aseptically prepared before use. Specifically, forceps were autoclaved, then immersed in 70% ethanol for 5 min and rinsed with sterile ultrapure water. Tools were allowed to air dry on a clean paper towel. Between each tick sample, the tools were immersed in a 25% bleach solution for several minutes, then placed in 70% ethanol, followed by immersion in ultra-pure water. After this process, the tools were allowed to air dry or were gently wiped with a sterile paper towel (https://ehs.stanford.edu/reference/comparing-different-disinfectants).

All the Petri dishes were wiped or sprayed with 70% ethanol and allowed to air dry. In certain cases, glass Petri dishes could be used to avoid damage by pointed forceps. A LEICA M205FA fluorescence stereomicroscope equipped with Fluocombi (LEICA, Wetzlar, Germany) was used for image acquisition.

### Approach

#### Tick rearing

For all experiments, batches of unfed *A. variegatum* ticks (larvae, nymphs, and adults) were reared as a colony for more than 13 years at the CIRAD animal facility in Guadeloupe. Larvae and nymphs were placed on the ear side of a naïve creole goat in a Mölnlycke Tubifast green line bandage, and secured with Kamar adhesive (Vital Concept, Loudeac, France). Adults, on the other hand, were placed on the goat’s flanks. Larvae were collected and allowed to molt in a container maintained at room temperature and 70–80% relative humidity. Subsequent to ecdysis, 10 nymphs were used for dissections after a brief pre-feeding period (approximately 20 days) during which they developed to a sufficient degree of maturity. In the case of adults, after completing feeding and detachment, 5 repleted females were collected and were allowed to undergo their pre-oviposition period (approximately 10 days) before being used for dissection. In total, 20 semi-engorged females were collected after a period of 7 days after the onset of their feeding, following several copulations and after approximately 12 days during reproductive physiological processes. However, in the case of engorged and sexually mature males, a total of 20 male ticks were manually removed from the goat after approximately 28 days of feeding and were dissected.

#### Tick preparation

Prior to dissection, ticks were washed twice for 30 s in ultrapure water and once in PBS in three 0.5 mL Eppendorf microtubes to remove impurities on the tick’s cuticle. During the washing process, the tubes containing the ticks were gently inverted by hand. As *A. variegatum* ticks are rapid crawlers, they could optionally be cooled on ice to slow them down. Following the washing step, the ticks were completely dried on Kimtech paper towels (Kimberly-Clark, Nanterre, France) before being immobilized for dissection.

**Figure 1:**
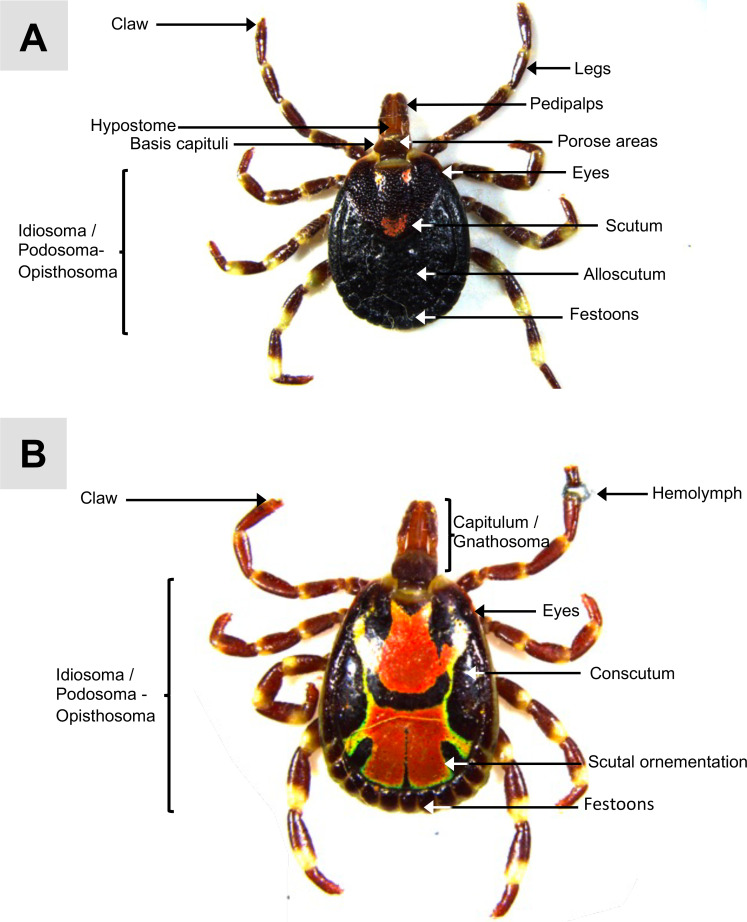
Anatomical description of the dorsal surface of unfed adult *A. variegatum* ticks mounted on double-sided adhesive tape. (A) Dorsal view of unfed *A. variegatum* female (0.78×). (B) Dorsal view of unfed *A. variegatum* male, with its first leg cut to allow hemolymph collection (0.78×).

Fixation of the tick was performed by immobilizing a single tick, with its ventral side down, on a piece of double-sided adhesive tape within a Petri dish (Fig. 1). To ensure fixation of the tick, all four legs were extended and secured to the double-sided tape with tweezers. For nymphs and unfed adults, multiple ticks could be fixed in a single Petri dish. Once immobilized and incised, a few drops of sterile, filtered Dulbecco’s Phosphate Buffered Saline (PBS) 1× were applied to cover the entire tick body. This prevented desiccation of the tick’s tissues and facilitated dissection by swelling the internal organs.

#### Hemolymph and saliva collection for research purpose

It is possible to collect saliva or hemolymph cells for further research studies. Our study did not focus on *A. variegatum* hemocytes or saliva, even though these are critical to vector competence.

**Figure 2:**
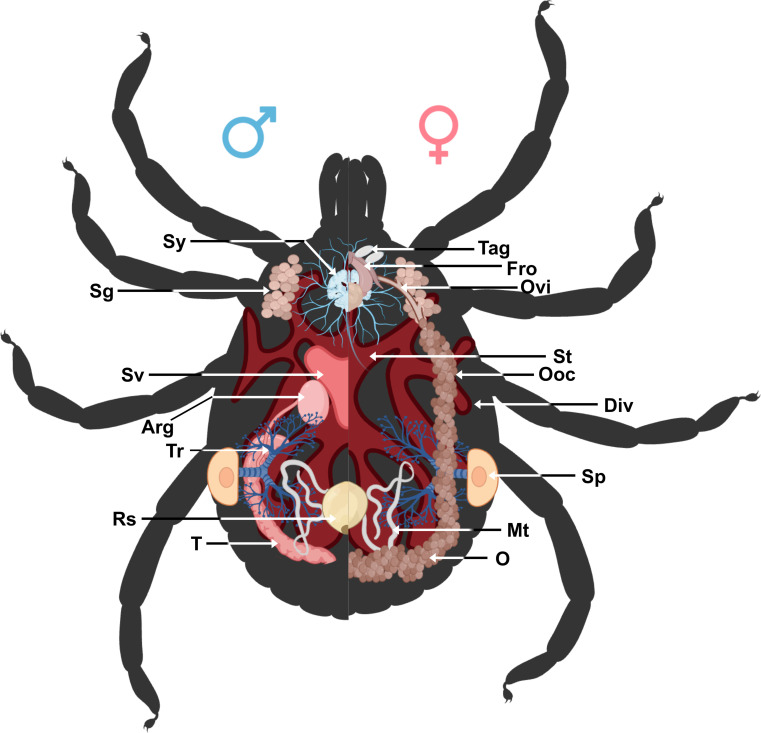
Schematic representation of the internal structure of hard ticks. Arg: accessory reproductive glands; Div: diverticulum; Fro: female reproductive organs; Mt: Malpighian tubules; O: ovary; Ooc: oocytes; Ovi: oviduct; Rs: rectal sac; Sg: salivary glands; Sp: spiracular plates; St: stomach; Sv: seminal vesicle; Sy: synganglion; T: testes; Tag: tubular accessory glands; Tr: tracheae.

To collect saliva, semi-engorged ticks that had been removed from animals or artificially fed were used to obtain larger amounts of saliva. After washing the tick as described above, it was immobilized on double-sided tape placed in the upper part of a Petri dish, with its ventral surface facing up to expose its mouthparts, including the rostrum and palps (Fig. 2). Another piece of double-sided tape was placed near the tick’s basis capitula on the edge of the Petri dish. A capillary tube was pressed between the hypostome and palps, ensuring the pedipalps remained outside the capillary tube. A solution of pilocarpine (20 mg/mL) was prepared, and 50 μL was gently injected into the tick’s stigmata or spiracles (Sp) (Fig. 2) using a Hamilton syringe. Salivation was observed, and the saliva was collected using a pipette tip inserted into a micropipette. If the tick did not salivate after 10 min, an additional 20 μL of pilocarpine was injected. The saliva should be stored at −80 °C for further research analysis or use.

**Figure 3:**
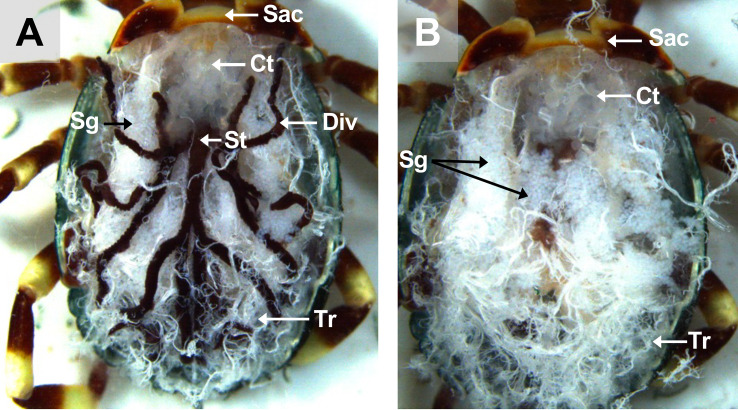
Dorsal view of an unfed *A. variegatum* female with its internal cavity exposed. (A) Scutum dissection: dorsal view of an unfed *A. variegatum* female with the scutum removed and immersed in PBS (1.6×). (B) Midgut dissection: Dorsal view of an unfed *A. variegatum* female with the midgut removed (3.2×). Ct: connective tissue; Div: diverticulum; Sac: **scutum**/alloscutum/conscutum (in the female tick); Sg: salivary glands; St: stomach; Tr: tracheae.

**Figure 4:**
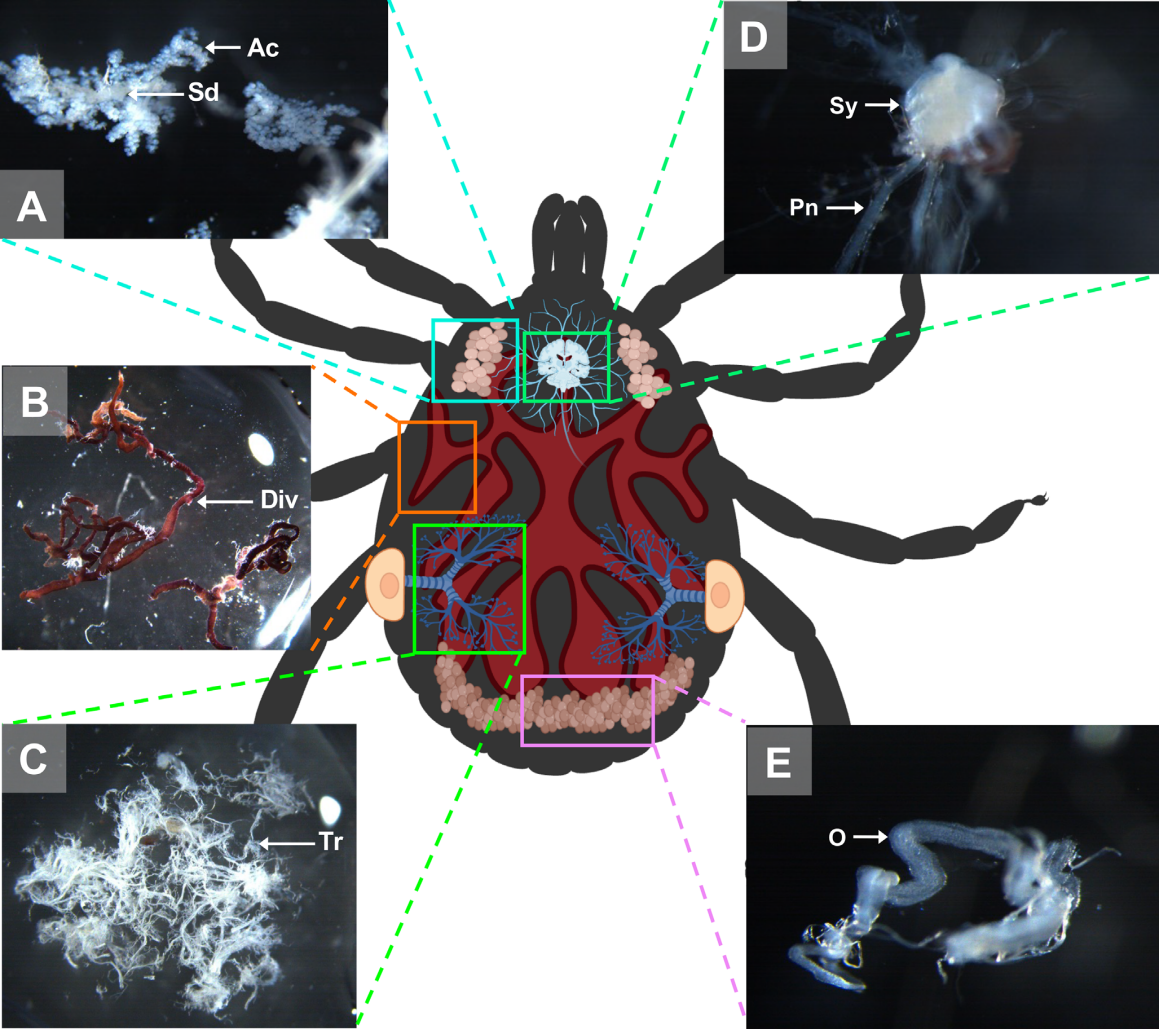
Schematic representation of an *A. variegatum* female and its organs. (A) Salivary glands dissection: close-up view of the salivary glands of an unfed *A. variegatum* female (3.2×). (B) Midgut dissection: close up view of the gut of an unfed *A. variegatum* female (1.25×). (C) Tracheae dissection: close-up view of the tracheae of an unfed *A. variegatum* female (1.25×). (D) Synganglion dissection: close-up view of the nervous system of unfed an *A. variegatum* female (5×). (E) Reproductive system dissection: close-up of the ovary of an unfed *A. variegatum* female (5×). Ac: acini; Div: diverticulum; Pn: peripheral nerves; Sd: salivary duct; Sy: synganglion; Tr: tracheae.

For hemolymph collection, the same procedure was used to wash the external structure of the tick. The ticks were then fixed on a double-sided tape in the same manner as for the dissection. To collect the hemolymph, the first two legs of the tick were fully extended. Then, the most distal joint of the tick’s tarsus was cut with a scalpel, and the drop was collected with a micropipette through a microcapillary tube (Fig. 1B). For larger volumes of hemolymph, several legs may be amputated and the tick’s body was gently squeezed to promote drainage. The collected hemolymph must be stored correctly to facilitate further studies planned.

### Tick general dissection

#### Dissecting the external structure: scutum/alloscutum/conscutum removal

**Figure 5:**
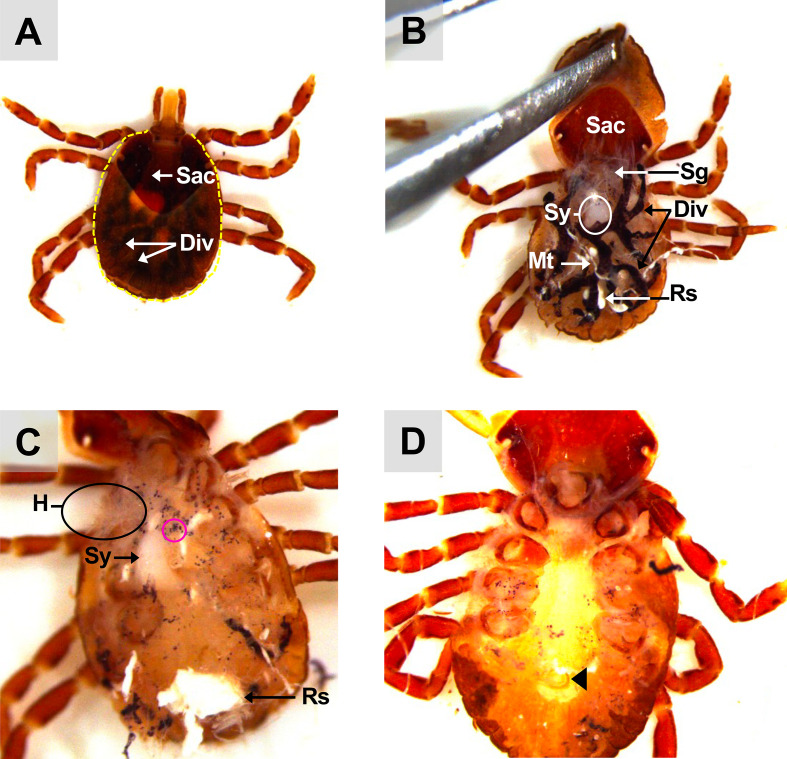
Dorsal view of an unfed *A. variegatum* nymph. (A) Dissection of the scutum: dorsal view of an unfed *A. variegatum* nymph immersed in PBS, showing a clean incision (yellow dotted line) around the idiosoma (2.5×). (B) Removal of the scutum: dorsal view of an unfed *A. variegatum* nymph immersed in PBS, with the conscutum lifted using forceps (2.5×). (C) Dissection of the midgut: *A. variegatum* nymph with the entire gut removed and the scutum cut off (3.2×). (D) Unfed *A. variegatum* nymph with the salivary glands, midgut, tracheae and Malpighian tubules removed (4×). Div: diverticulum; H: heart; Mt: Malpighian tubules; Rs: rectal sac; Sac: **scutum/ alloscutum**/conscutum (here, in the nymph tick); Sy: synganglion; pink circle: hematin granules released after gut tear; black triangle: anal aperture.

Once the tick was firmly immobilized, the scutum/alloscutum/conscutum (Sac) was removed. A sterile no. 11 blade was used to remove the Sac. This sterile blade was used to incise the dorsal cuticle at the distal part of the basis capitulum. A microscalpel was required to dissect ticks, particularly nymphs, to avoid damaging internal organs. The cutting step continued around the tick’s Sac, passing above the festoons, and reaching the opposite side precisely (Fig. 5A). Special care was taken to keep the internal cavity intact while cutting the dorsum of the tick by bypassing the spiracular plates, which are connected to the internal organs.

**Figure 6:**
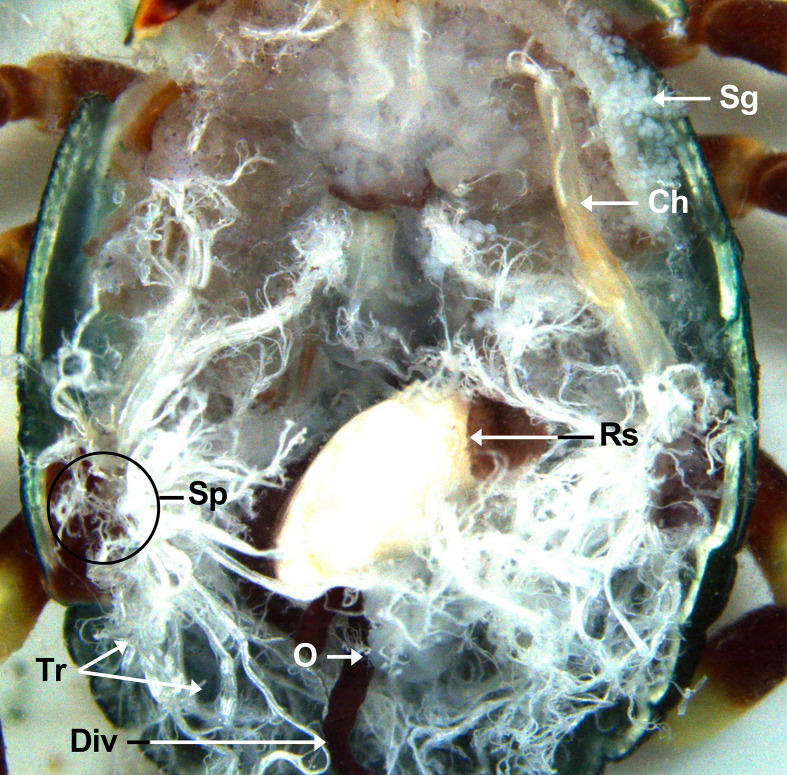
Dorsal view of an unfed *A. variegatum* female with midgut and main salivary glands removed (2×). Ch: chelicerae; Div: diverticulum; O: ovary; Rs: rectal sac; Sp: spiracular plates; Tr: tracheae.

Following incision of the Sac, two dissecting forceps (Entomopraxis, Barcelona, Spain) were used to remove it. Simply put, one forceps held the tick’s body on the double-sided tape, while the other forceps carefully lifted the Sac away from the rest of the body. Once the cuticle was lifted, all of the thin white tubules and attached muscles were removed from the Sac to gain complete access to the tick’s internal anatomy (Fig. 3A). The dorsum was either glued to the double-sided tape or cut off completely from the tick. At this point, the small white tubes with a spider’s web shape are designated as tracheae (Tr).

#### Dissecting the respiratory system: tracheae removal

**Figure 7:**
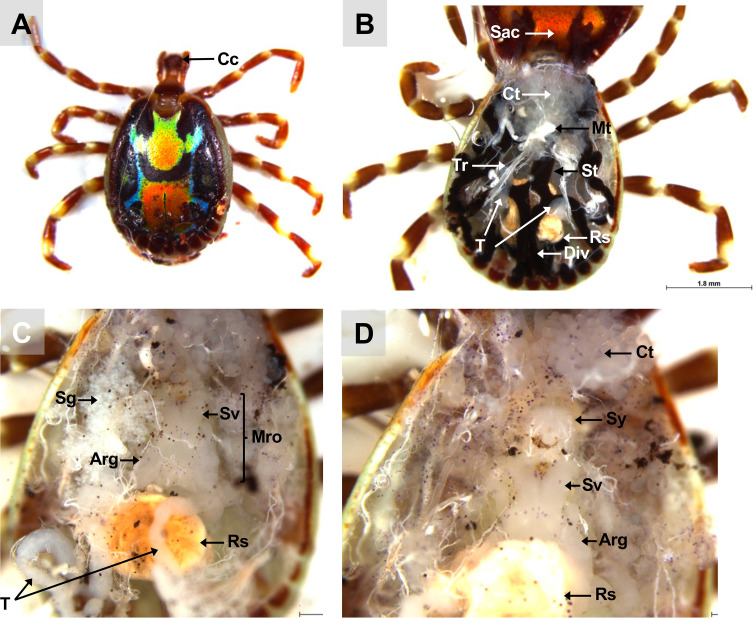
Dorsal view of a fed and mated *A. variegatum* male. (A) Dorsal view of a fully repleted *A. variegatum* male with a rounded conscutum (1.25×). (B) Scutum dissection: dorsal view of a fed *A. variegatum* male with folded conscutum, immersed in PBS (1.6×). (C) Midgut dissection: *A. variegatum* male with the entire gut removed (1.6×). (D) Salivary glands dissection: *A. variegatum* male with the paired salivary glands removed (2.5×). Arg: accessory reproductive glands; Cc: cement cone; Ct: connective tissue; Div: diverticulum; Mro: male reproductive organs excluding testes; Mt: Malpighian tubules; Rs: rectal sac; Sac: scutum/alloscutum/**conscutum** (in the male tick); Sg: salivary glands; St: stomach; Sv: seminal vesicle; Sy: synganglion; T: testes; Tr: tracheae.

Once the attached muscles and connective tissue were carefully removed from the ventral part of the dorsum, the entire tick cavity became visible (Figs. 3A, 5B, 7B). To release the internal organs, two dissecting forceps were gently used to grasp the tracheae at the spiracular plates (Sp) between the third and fourth pairs of legs on each side of the tick (Figs. 6, 10A).

**Figure 8:**
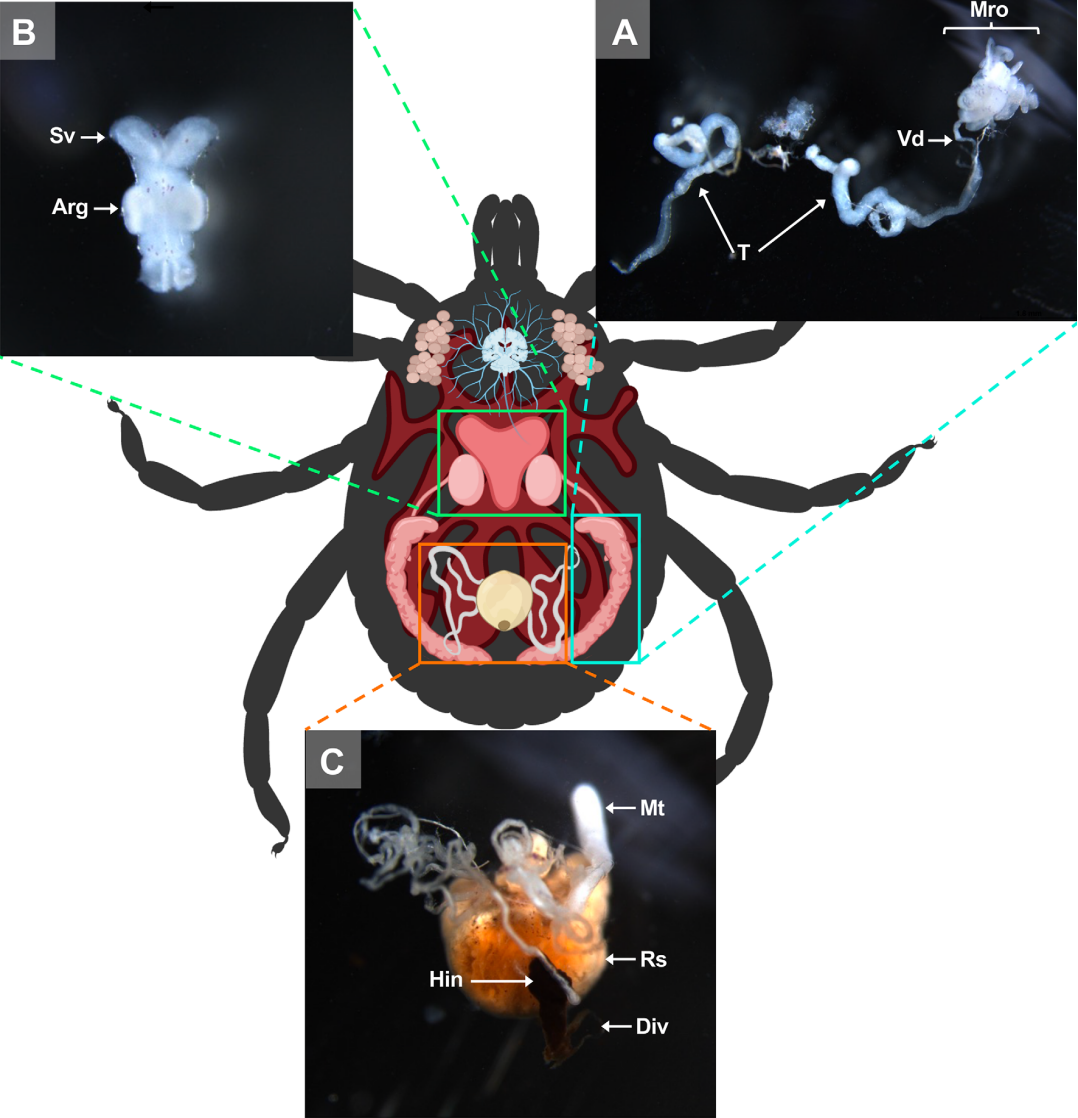
Schematic representation of an *A. variegatum* male and its organs. (A) Reproductive organs dissection: highlighting the organs of the male reproductive system (1.6×). (B) Reproductive organs dissection: close-up view of accessory reproductive glands (2.5×). (C) Close up of the excretory system (2.5×). Arg: accessory reproductive glands; Div: diverticulum; Hin: hindgut; Mro: male reproductive organs excluding testes; Mt: Malpighian tubules; Rs: rectal sac; Sv: seminal vesicle; T: testes; Vd: vasa deferentia.

**Figure 9:**
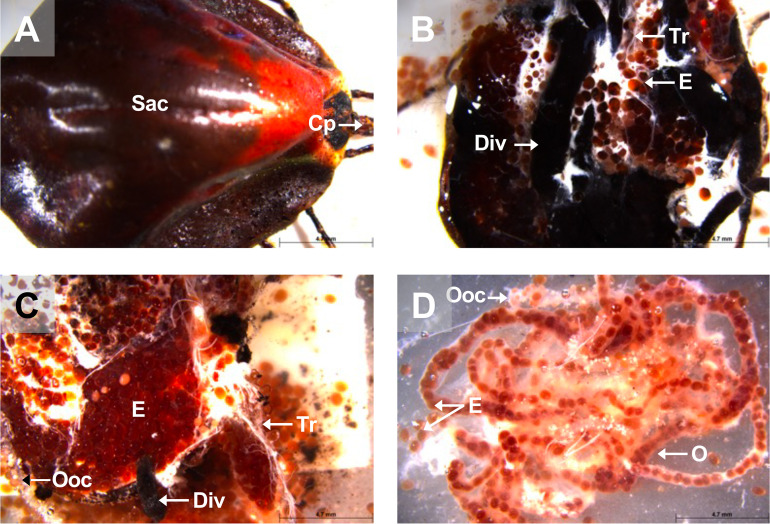
View of a fully repleted *A. variegatum* female. (A) Dorsal view of a fully engorged *A. variegatum* female (0.78×). (B) Dorsal view of an ovipositing *A. variegatum* female with scutum removed (0.78×). (C) Dorsal view of *A. variegatum* with thousands of eggs expelled from the oviducts (0.78×). (D) Close up of oviducts full of eggs (0.78×). Cp: capitulum; Div: diverticulum; E: eggs; Ooc: oocytes; O: ovary; Sac: **scutum/alloscutum/**conscutum (in the female tick); Tr: tracheae.

**Figure 10:**
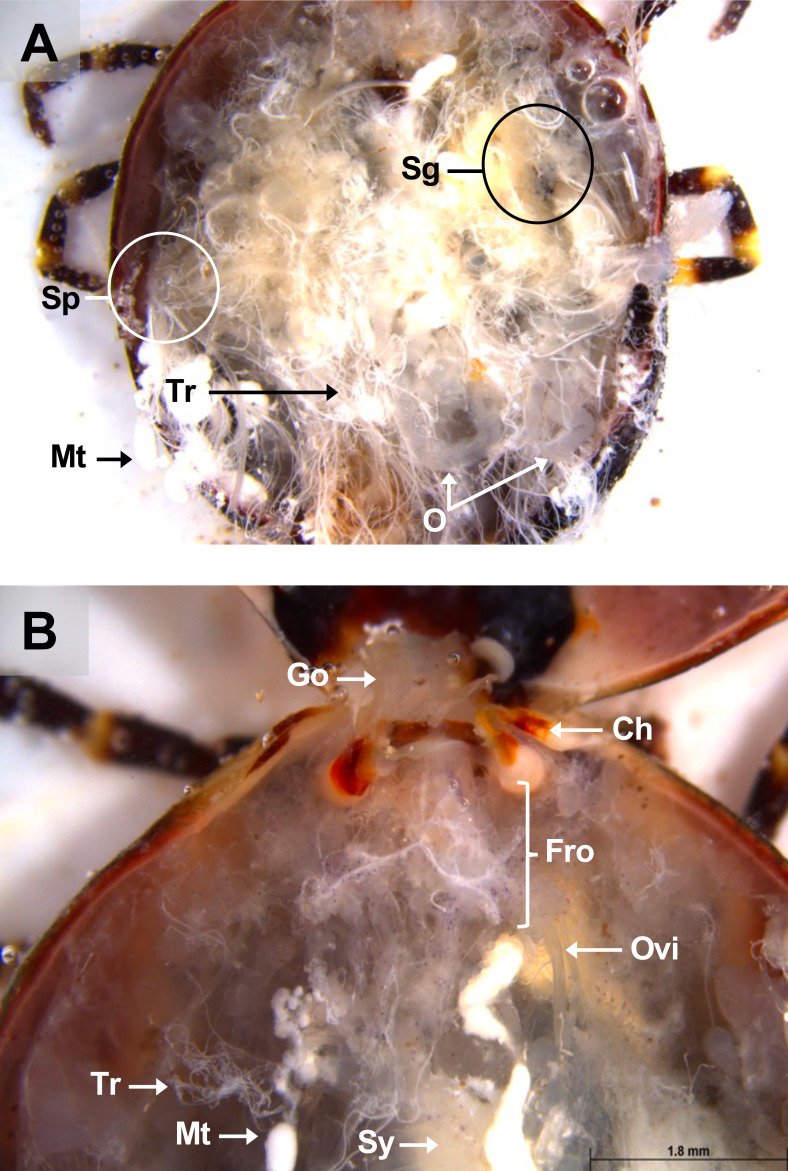
Dorsal view of a partially fed *A. variegatum* female. (A) Dorsal view of a semi-engorged female *A. variegatum* female with the midgut removed (1×). (B) Dorsal view of a female *A. variegatum* with a close-up of reproductive system (2×). Ch: chelicerae; Fro: female reproductive organs excluding ovary and oviducts; Go: Gené’s organ; Mt: Malpighian tubules; O: ovary; Ovi: oviduct; Sg: salivary glands; Sp: spiracular plates; Sy: synganglion; Tr: tracheae.

In some instances, when dissecting feeding adult female ticks, the tracheal trunks at the base of the spiracular plates were observed to be denser. If difficulties arose, the spiracular plates were incised, and the tracheal trunk carefully extracted with the forceps. After removal of the tracheae, the salivary glands (Sg) became more prominent.

#### Dissecting the digestive system: midgut removal

The midgut stomach (St) was withdrawn from the tick body by grasping the junction of the esophagus and stomach with one dissecting forceps and retracting the midgut with another pair of forceps. The esophagus is a narrow, elongated tube that runs anteroventrally from the synganglion to the anterior part of the stomach.

**Figure 11:**
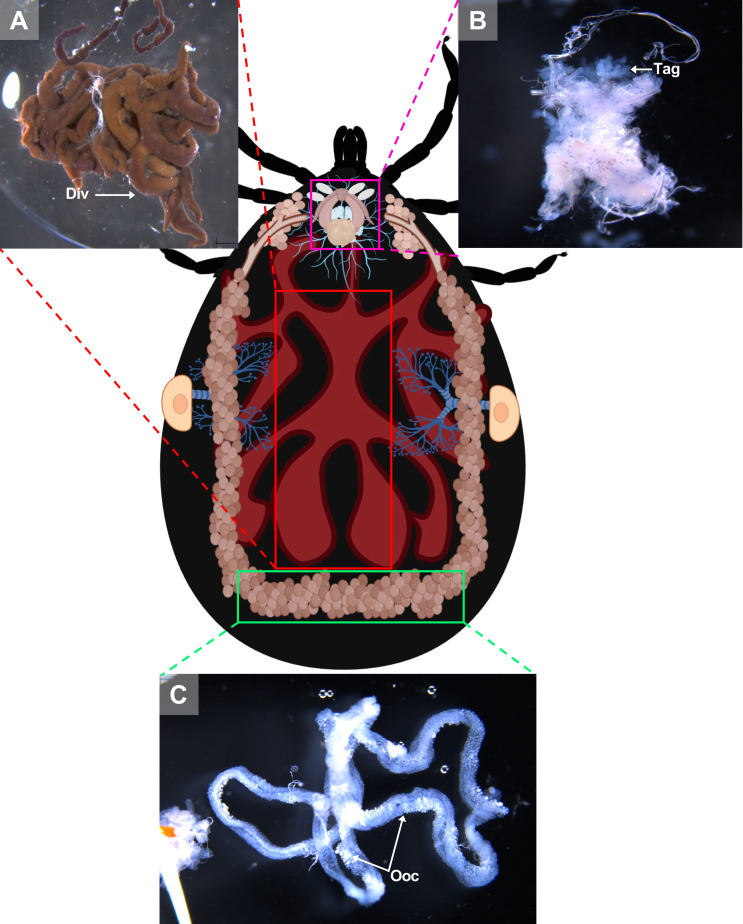
Schematic representation of an engorged *A. variegatum* female and organs. (A) Close-up of the midgut of a partially fed female *A. variegatum* female (0.78×). (B) Close up of reproductive organs with accessory glands (2×). (C) Close-up of ovary with oocytes (1.6×). Div: diverticulum; Ooc: oocytes; Tag: tubular accessory glands.

Subsequently, the diverticula (Div) of the midgut could be gently pulled away from the rest of the organs without damaging them, ensuring that their digestive contents were not discharged (Figs. 3B, 4B, 10A, 11A). Since the midgut is elastic, it can be easily removed while ensuring that it remains intact. The midgut was rinsed in three successive pools of sterile PBS with aseptic dissecting forceps if analysis was required [30]. After removal of the midgut, the salivary glands (Sg) and the synganglion (Sy) became more visible (Fig. 3B, 5C, 7C).

Two pairs of larger white tubes located laterally to the midgut, represent the Malpighian tubules (Mt), which are clearly visible. The Malpighian tubules, which are part of the tick excretory system, are connected to the posterior of the intestines, known as the hindgut (Hin), and the rectal sac (Rs).

#### Dissecting the digestive and the nervous system: salivary glands and synganglion removal

Two dissection forceps were employed to collect the salivary glands. One forceps was used to grasp the principal salivary duct at the base of the chelicerae (Ch) at the anterior upper end, while the other was used to gently pull out the salivary glands. As with the midgut, the salivary glands were rinsed in three baths of sterile PBS prior to collection and cleared of any residual tracheae and connective tissue (Ct) (Fig. 4A). The same procedure was applied to remove the salivary gland on the opposite side. The size of the salivary glands varies with the feeding status of the ticks, becoming larger in half-fed ticks and atrophic in fully-fed ticks. The glands could be rinsed in three pools of PBS on either a Petri dish or a sterile glass slide, using aseptic dissecting forceps.

Once the salivary glands were removed, the heart became visible especially in nymphs (Fig. 5C). The heart (H) lies in the dorsal portion along the mid-sagittal axis and is connected anteriorly with the aorta, and leads to the synganglion (Fig. 5C). Given the fragility of the synganglion, its removal requires precision. One forceps can be used to grasp the whitish neuronal extensions located on its lateral side (Fig. 4D).

#### Dissecting the reproductive organs: testes and ovaries removal

Following the removal of the salivary glands, the tick’s reproductive system, particularly in males, may become more apparent (Figs. 7C, 7D). It is important to note that the reproductive system only develops at the adult stage. Consequently, the nymph lacks both reproductive organs and a gonopore, which are essential for reproduction (Figs. 5C, 5D).

#### Dissection of males: testes

The male reproductive system includes the genital opening (gonopore), testes (T), vasa deferentia (Vd), ejaculatory duct, seminal vesicle (Sv), and accessory reproductive gland complex (Arg). Since the internal anatomy of *A. variegatum* males is simpler than that of females, isolating the organs is relatively easier (Figs. 3A, 7B). After removing the trachea and the midgut, the reproductive system (Mro) becomes exposed, particularly the testes, which occupy significant space (Figs. 7C, 7D). Using dissecting forceps, the ejaculatory duct can be gently pulled out of the tick’s body, bringing the entire reproductive system with it (Figs. 8A, 8B).

#### Dissection of females: ovaries

Gené’s organ (Go), a specialized structure found only in female ticks, is located in the anterior region of the cavity, just above the basis capituli. This structure facilitates coating the eggs with a wax-like substance during oviposition.

During dissection, with the ventral surface of the tick facing downward, Gené’s organ is located dorsally, above the synganglion, while the female reproductive apparatus is positioned ventrally to the synganglion. For better access to Gené’s organ, the capitulum can be removed with a surgical blade or scissors (Fig. 10B).

An incision should be made horizontally beneath the basis capitulum, just above the camerostome, to avoid damaging the organ. Once the connective tissue and residual tracheae are cleared from the tick’s internal cavity, Gené’s organ becomes visible. Using dissecting forceps, Gené’s organ can be extracted from the camerostomal opening by firmly grasping it. It should be noted that the tubular accessory glands of Gené’s organ do not fully develop until the female has completed a blood meal (Figs. 10B, 11B). Following the removal of the trachea and the midgut, the oviducts (Ovi) leading to the ovary (O) in the posterior region, and the vagina, located ventrally to the synganglion, should become visible (Fig. 10B).

**Figure 12:**
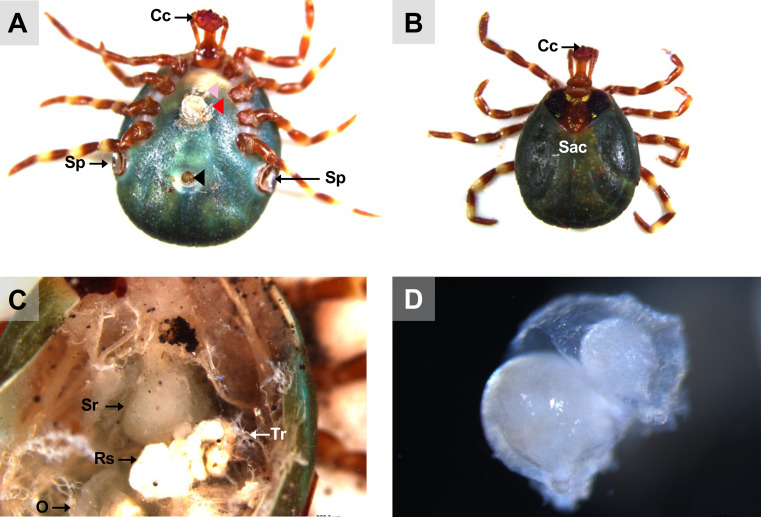
View of a mated *A. variegatum* female. (A) Ventral view of a semi-engorged *A. variegatum* female with an ectospermatophore attached near to its gonopore shortly after copulation (0.78×). (B) Dorsal view of a partially fed *A. variegatum* female with a round alloscutum (0.78×). (C) Dorsal view of an endospermatophore contained in the seminal receptacle of a semi-engorged *A. variegatum* female (2.5×). (D) Close-up of the endospermatophore dissected a few minutes after the female mated with a male (2.5×). Cc: cement cone; O: ovary; Ooc: oocytes; Rs: rectal sac; Sac: scutum/ alloscutum/conscutum (in the female tick); Sr: seminal receptacle; Sp: spiracular plates; Tr: tracheae; black triangle: anal pore; pink triangle: gonopore; red triangle: ectospermatophore.

The vagina was then grasped with dissecting forceps, and the entire female reproductive apparatus was carefully removed from the internal cavity (Fig. 11C). This apparatus includes the bipartite vagina, seminal receptacle, uterus, accessory glands, oviducts, and ovary. It should be noted that these components have not been further differentiated. Similar to males, the female genital opening is located ventrally relative to the synganglion (Fig. 12A).

### Practical notes

During dissections, care must be taken to avoid tissue tearing, especially for fragile organs like ovaries and synganglion. Using fine, aseptic tools and working under a stereomicroscope significantly reduces the risk of damage. Adhesive tapes and clean dissecting surfaces are essential to maintain tissue integrity and minimize contamination. For less experienced researchers, it is advisable to practice dissections on nymphal or male ticks, which have simpler anatomy, before attempting engorged females. If tissues are intended for downstream analyses, such as transcriptomics or proteomics, meticulous handling and immediate storage in appropriate solutions (e.g., phosphate buffer saline (PBS) or RNAlater) are critical to preserve RNA or protein quality.

## Results and discussion

### External morphology and key taxonomic features

#### *A. variegatum* ticks are characterized by a distinctive external scutum ornamentation

Proper dissection of *A. variegatum* necessitates familiarity with its external morphology. The tick body, similar in structure to that of parasitiform acarines such as mites is divided into two main regions: the gnathosoma (capitulum) and the idiosoma (body region) (Fig. 1). The capitulum, considered to be the “head”, is located in the anterior part and bears the mouthparts – the chelicerae, hypostome, and palps – but does not house the eyes or brain, which are located within the idiosoma.

The basis capituli of *A. variegatum* is rectangular to oval, as observed in *A. parvitarsum* and *A. lepidum* [3, 25]. Additionally, the basis capituli, located in the capitulum, bears two paired structures known as porose areas that are nearly circular in shape in *A. variegatum* females (Fig. 1A). The porose areas of the female *A. variegatum* tick are observed consistently with those arranged in *A. varium* or in the *A. parvitarsum* tick, which belongs to the *A. maculatum* group [25, 47]. In contrast, a greater interdistance between the porose areas was observed in *A. maculatum*, *A. tigrinum*, and *A. triste*.

Of note, *A. variegatum* is considered a large tick, ranging from 6–7 mm when unfed to up to 30 mm in repleted females, similar in size to *A. crissum* and *A. gaeyi* [38, 71]. The size of the *A. variegatum* hypostome is of particular concern as it has been observed to cause further damage to the skin of its host [35]. These ticks are classified within the Longirostrata, distinguished by their elongated mouthparts, a feature shared by species such as *A. varium* and *A. coelebs* [71]. The hypostome of *A. variegatum* ticks is characterized by its elongated cylindrical form, situated at the same level as the two paired palps, a feature shared by the *A. sculptum* and *A. patinoi* tick species [40].

The idiosoma is further divided into the anterior podosoma, which includes the genital gonopore and four pairs of legs, and the posterior opisthosoma, which contains spiracular plates and the anal pore [64]. Additionally, the idiosoma includes the walking legs. As ixodid ticks (hard-bodied ticks), *A. variegatum* displays a teardrop-shaped body with a dorsal shield, or scutum [19].

In males, the scutum (conscutum) fully covers the dorsum, restricting the ingestion of large quantities of blood during blood feeding. *Amblyomma variegatum* males exhibit unique ornate patterns in bright orange colors and dark hues, a characteristic also found in other African species such as *A. lepidum*, *A. hebraeum*, *A. pomposum*, and *A. eburneum* (Fig. 1B, 7A) [3, 63].

In contrast, the scutum of adult females, nymphs and larvae, does not cover the entire dorsum. The female scutum consists of a large subtriangular plate near to the capitulum, while the remaining two-thirds of the idiosoma is made up of a less sclerotized plate, the alloscutum (Figs. 1A, 9A). In contrast to the distinctive ornamentation, shape, and shiny colors exhibited by *A. variegatum* males, *A. variegatum* females are distinguished by a less specific figure (Fig. 1). The scutum of the female exhibits bright colors that evoke the conscutum of the male. However, given that the scutum is smaller in size compared to the rest of the body, several species of *Amblyomma* ticks exhibit remarkably similar patterns [40]. During feeding, the alloscutum stretches to accommodate a significant increase in volume, allowing females to reach up to 100 times their unfed weight (Fig. 9A).

Furthermore, the alloscutum of larvae and nymphs tends to enlarge as they ingest blood. *Amblyomma variegatum* nymphs’ overall morphology is similar to that observed in *A. hebraeum* nymphs [7]. *Amblyomma variegatum* nymphs are comparable to females, except that they exhibit a brown coloration, instead of black, and a less pronounced pattern of alternating-colored legs (Figs. 1A, 5A). Lateral to the scutum/conscutum, two hemispherical, dark-colored orbital eyes can be observed (Fig. 1, 5A). *Amblyomma variegatum* possess eyes that are distinctly convex, displaying notable similarities to those of *H. impressum* or *H. dromedarii* when compared with *R. sanguineus*, where the eyes are not distinctive [1, 2].

The locomotor system includes four pairs of legs in adults and nymphs, and three in larvae. Each leg is composed of six podomeres – coxa, trochanter, femur, patella, tibia, and tarsus – arranged proximally to distally on the podosoma [65]. The legs of ticks are highly flexible, owing to elastically articulated segments with bright colors in *A. variegatum* ticks (Fig. 1). In the *A. variegatum*, the coloration of the legs exhibits a pattern alternating between brown and pale rings. This trait is also observed in some *Hyalomma* species [2]. *Amblyomma variegatum* legs are slightly slender in comparison with the size of its body, in contrast to the bulbous legs exhibited by *Margoropus* ticks [72]. At the posterior end of the opisthosoma of male and female ticks, as well as nymphs, several festoons are present, separated by a marginal groove (Fig. 1). In certain species of the *Amblyomma* genus, males have been observed to exhibit enamel displayed as patches on individual festoons [72]. However, *A. variegatum* males do not exhibit enamel on their festoons, in contrast to *A. hebraeum*, *A. lepidum*, and *A. eburneum* [3, 63].

### Internal Anatomy of *A. variegatum*

#### *Amblyomma variegatum* females have a well-developed tracheal system

The tracheae are part of the respiratory system of ticks and facilitate gas exchanges through the body and organs of the tick [75]. The tracheal system of the tick is extensive and opens externally through two lateral spiracular plates [66]. It is noteworthy that there is considerable variation in the structure of the respiratory system among ticks, particularly with regard to the morphology of the spiracles. In *A. variegatum*, the spiracular plates of the female specimen are considerably larger than those of the male specimen. This is due to the fact that females have a larger body size and distinct physiology. These physiological mechanisms, including the digestion of large blood meals, excretion, and egg production, require larger spiracular plates to ensure sufficient surface area for energy exchange via gas exchange [8]. Thus, the tracheal system of *A. variegatum* females is much denser than that of the males, complicating its dissection (Figs. 3A, 7B). In contrast, larval tracheae are rudimentary and enable passive gas diffusion across the integument [12].

#### The digestive system of *A. variegatum* ticks is larger than that of other tick species

In ixodid ticks, the pharynx is an elongated organ situated on the ventral surface of the basis capitulum. It facilitates the passage of ingested host blood through the food channel to the esophagus and midgut [64]. The gut extends throughout the entire body region of the tick and represents its largest organ. The entire tick gut includes the midgut, with a central portion, the stomach, which is connected to several dark brown, spider-shaped diverticula (Figs. 2, 3A, 5B, 7B). During the tick’s engorgement phase, the midgut-diverticula are filled with host blood to be digested (Fig. 11A). Due to the considerable size of the midgut, other internal organs may be displaced or compressed (Fig. 9B) [64]. This phenomenon is particularly salient for *A. variegatum* females, as they possess a larger body size and consume greater quantities of blood compared to other tick species. Consequently, withdrawal of the gut, without causing damage to it, is a complex procedure. The hindgut and the Malpighian tubules are key components of the tick’s excretory system, playing vital roles in maintaining homeostasis and excreting guanine-rich wastes while transporting them to the rectal sac [62]. In ticks, nitrogenous excreta from the Malpighian tubules and undigested waste from the midgut are concentrated in the rectal sac and eliminated through the anus via a short rectum (Figs. 5C, 7C) [62]. These connections are situated beneath the intestinal tissue. In Fig. 8C, the excretory system is faintly visible once it has been extracted from the cavity.

Tick salivary glands are complex, multi-lobed organs that serve multiple functions during both the tick’s parasitic and non-parasitic periods on the host [14]. The salivary glands are paired structures located in the anterolateral regions of the tick’s body (Fig. 2). The salivary glands of female ixodid ticks consist of clusters of grape-shaped alveoli, called acini, which are categorized into three morphotypes: type I, type II, and type III [61]. *Amblyomma variegatum* tick salivary glands appeared slightly more opaque compared to those of *R. microplus* [18] and *I. scapularis* ticks [64]. As observed in partially engorged but interrupted *A. variegatum* females, the salivary glands exhibited greater translucency compared to those of unfed females (Figs. 4A, 10A). This observation is consistent with the findings of a recent study on *R. haemaphysaloides*, which reported the degeneration of salivary glands in engorged female ticks after 7 days of feeding [73]. The hypothesis that the initiation of autolysis of salivary glands was associated with a peak in hemolymph ecdysteroid concentration was proposed [37]. Furthermore, evidence suggests that the release of ecdysteroids during the post-engorgement period triggers both salivary gland degeneration and ovarian development [28]. These findings support the need for careful handling during saliva collection. In this study, tick saliva was collected subsequently to the injection of pilocarpine through tick spiracles. However, it has been presumed that pilocarpine is active in certain bioassays [53]. Consequently, a cautious interpretation of the results obtained from tick saliva samples treated with pilocarpine is imperative.

#### The nervous system of ixodid ticks, including *A. variegatum,* appears to be significantly similar

The salivary glands are innervated by branched peripheral nerves arising from the synganglion, the central nervous system of ticks (Fig. 2). This organ performs several essential functions in ticks, including movement control and stimulus processing [54]. The synganglion is located in the ventro-anterior region, posterior to the midgut stomach, and is composed of multiple ganglia [41]. The synganglion is a translucent, heart-shaped structure linked to a multitude of peripheral nerves that innervate various parts of the tick body [64]. A comparison of the anatomical structure of *A. variegatum* synganglion and *R. microplus* or *I. scapularis* reveals significant similarities [18, 67, 68].

As part of an open circulatory system, the pumping action of the heart and the arterial vessels facilitates circulation within the hemocoel (Fig. 5C) [12]. The heart appears pentagonal in systole and spherical in diastole [18]. In comparison with nymphs, the internal structure of adult *A. variegatum* ticks is more complex, which hinders the visualization of the heart.

#### *Amblyomma variegatum* male testes exhibit a tubular and elongated shape

The male reproductive system includes the gonopore, testes, vasa deferentia, ejaculatory duct, seminal vesicle, and accessory gland complex. The male gonopore is located in the ventroanterior region of the body, ventral to the synganglion, and opposite the anal aperture [58].

The male posterior region is composed of a pair of tubular and elongated testes, positioned dorsolaterally within the opisthosoma. These are connected to the accessory reproductive glands by the vasa deferentia (Figs. 6E, 6F) [6, 57]. The testes serve as the primary site of spermatogenesis, which begins during the nymphal stage. The appearance of tick testes may vary slightly even within a single species, depending on the nutritional and reproductive status of the individual [45]. In metastriate ticks, the testes are linked by an extremely thin strand of filamentous tissue and taper into the vasa deferentia. The testes are located laterally to the rectal sac [43]. *Amblyomma variegatum* males possess a reproductive system that is morphologically similar to that of *R. sanguineus* [56]. However, a notable distinction emerges in the structure of the seminal vesicles, which exhibit a specific V-shape in *A. variegatum* male ticks, contrasting with the more elongated appearance observed in *R. sanguineus*. While both *A. variegatum* and *R. sanguineus* possess two pairs of testes, *O. rostratus*, by contrast, appears to lack seminal vesicles and presents a single horseshoe-shaped testis. Furthermore, a comparison of *R. microplus* males and *A. variegatum* males reveals significant differences in the morphology of their testes. Specifically, *A. variegatum* males possess testes that are thinner and less pronounced in the opisthosoma compared to *R. microplus* males, which have more rounded and translucent testes [68]. Furthermore, the testes and the accessory glands complex of fed male *A. variegatum* are slightly larger than those of *A. hebraeum* [74]. The vasa deferentia represent the narrowing of the paired testes at their anterior ends, joining in the anterior region to form a single duct called the vas deferens or seminal vesicle. As with the testes, the shape and the size of the vasa deferentia depend on the reproductive state of the tick and can vary from narrow, coiled tubes to enlarged, straight structures [43]. In accordance with observations made in other *Amblyomma* species, including *A. sculptum*, *A. aureolatum* and *A. triste*, the vasa deferentia are short, consistent with the characteristics observed in *A. variegatum*. As demonstrated by Sampieri *et al.* (2016) [58], *A. triste* possesses an elongated tissue linking the two testes, in contrast to *A. variegatum* which lacks this isthmus. Histologically, the vasa deferentia are indistinguishable from the common vas deferens. The seminal vesicle serves as the point of connection between the vasa deferentia and the ejaculatory duct.

The ejaculatory duct is a short, narrow tube located in the ventroanterior region, extending below the synganglion to the external gonopore [23]. The male accessory reproductive glands (Arg) play critical roles in all stages of the reproductive biology of the mated female, from the sperm deposition to oviposition [29]. For instance, male accessory glands stimulate engorgement in females and activate spermatozoa upon arrival at the female seminal receptacle [52]. This multi-lobed complex is located ventrally to the opisthosoma and connects to the ejaculatory duct on its ventral face and to the vasa deferentia on the dorso-lateral region [57].

Mated male ticks are capable of excreting a sac-like spermatophore that protrudes from their genital opening and delivering it with their mouthparts to the female’s gonopore (Fig. 12A) [34]. During mating, metastriate males appear to insert only their chelicerae into the female’s genital pore, leaving the hypostome and palps outside, barely in contact with the female’s surface [34, 49]. This mechanism ensures the successful transfer of the sac-like spermatophore protruding from the male genital opening (Figs. 12C, 12D).

#### The female tick (*A. variegatum*) reproductive organs are more challenging to observe under the microscope

The female genital system comprises the gonopore, similar to that in male, ovaries, a common oviduct or uterus, a bipartite vagina divided into a cervical and vestibular vagina, and a seminal receptacle (Sr). Additionally, females possess two accessory glands: tubular and lobular. In *A. variegatum*, anatomical visualization of the uterus and vagina proves particularly challenging.

As demonstrated in *A. cajennense* [20], *R. sanguineus* [42], and *R. microplus* [55], *A. variegatum* females possess a unique, tubular, horseshoe-shaped, and continuous ovary located in the posterior part of the opisthosoma (Fig. 4E). The tick ovary is a hollow organ filled with oogonia and primary oocytes at various early stages of development. The ovary of unfed *A. variegatum* females is slightly smaller than average and appears translucent. It seems to be surrounded by a tunica propria, as observed in *H. longicornis* (Fig. 6) [76]. A fold or longitudinal groove extends along the antero-dorsal surface of the ovary, containing primary oocytes internally at their earliest stage of development, and more advanced oocytes externally (Fig 11C) [21]. During feeding, the female’s oocytes tend to protrude into the hemocel, giving the ovary a grape-like shape. As demonstrated in *A. rotundatum*, we observed oocytes of different sizes at various developmental stages after the dissection of a semi-engorged female (Fig. 10A) [59]. As *A. variegatum* ticks are known to have a complex life cycle in the wild, they exhibit a high reproductive potential, with an oviposition capacity of up to 20,000 eggs. Here, an *A. variegatum* female was observed during the pre-oviposition period, indicating its imminent oviposition (Fig. 9). The tick’s entire body was found to be filled with mature eggs and oocytes at earlier stages of development. It is evident that the tick ovary is significantly larger in size in comparison to its unfed state, which is consistent with the large size of *A. variegatum* females during their blood meal on a host.

At each end of the ovary are whitish, paired, and folded oviducts (Ovi), which converge to form a common oviduct or uterus (Fig. 10B). The oviduct tends to stretch considerably during the passage of the eggs due to peristaltic contractions of the oviduct walls (Fig. 9D). The uterus connects to the cervical vagina via a short tube. As indicated in the extant literature, the fed *A. variegatum* female observed here, possessed two thin folded oviducts that lead to the female reproductive organs (Fig. 10B). These oviducts have a thin diameter, similar to those observed in *R. microplus* females [70].

The vagina consists of a posterior, broad, and short cervical region, as well as an anterior, narrow, and long vestibular region [21]. In addition to the uterus, the seminal receptacle, tubular accessory glands and the vestibular vagina converge in the cervical vagina (Fig. 11B). The vestibular vagina extends from the gonopore to the cervical vagina and opens externally to the gonopore via the vulva. In the female *A. variegatum*, the vagina situated in the podosoma is more complex to observe.

The seminal receptacle is a sac-like extension of the cervical region that serves as the storage site for spermatozoa after mating, facilitating the fertilization of eggs [44]. Final stages of spermiogenesis occur in the seminal receptacle of copulated females until full engorgement (Fig. 12C) [15]. This structure is positioned above the uterus and opens postero-dorsally into the cervical vagina [21]. In the present study, an ectospermatophore was observed to be adhered near the genital aperture of the *A. variegatum* female (Fig. 12A). The spermatophore of *A. variegatum* males appears larger than that of *R. microplus* [70]. *A. variegatum*, like argasid ticks, possesses an ectospermatophore with a bulb-shaped capsule associated with a short neck [26]. Additionally, an endospermatophore was observed within the seminal receptacle of the female (Fig. 12C). As previously documented, a sheath surrounding the endospermatophore was observed in *A. variegatum*, likely forming the capsule enclosing the sperm [27]. However, the literature also documents that the endospermatophore of ixodid ticks is monolobed with the formation of a unique capsule [26]. However, consistent with the observations made by Oliver (1974), who noted the presence of an additional small sphere, termed the “endospermatophore neck”, in proximity to the endospermatophore in *D. occidentalis* [46], we have observed a comparable structure in the spermatophore of *A. variegatum*.

Gené’s organ is unique to female ticks. It secretes a waxy substance that covers the oviposited eggs, protecting them from the environment. A peculiarity of this organ is its ability to evert itself from the camerostomal aperture, located between the posterior base of the capitulum and the anterior region of the scutum. Internally, this organ is situated in the anterior body region, below the capitulum, and consists of branched tubular glands that are highly developed in the engorged females (Fig. 10B). In this study, we observed that the female *A. variegatum* Gené’s organ exhibited a structural similarity to that of *R. microplus* females, thereby highlighting the conventional form of this organ [68].

The tubular (Tag) and lobular accessory glands are associated with the Gené’s organ and also contribute to the egg waterproofing during the oviposition period of the female. The tubular glands form the bulk of Gené’s organ and are located anteriorly within the hemocoel, in continuity with the horns (Fig. 11B). The paired tubular accessory glands open into the vagina at the dorsal boundaries of the cervical and the vestibular regions [13]. During oviposition, the lumen of each gland fills with a secretion that coats the egg surface as it passes through the vagina [21]. In contrast with *R. microplus*, the tubular accessory glands of *A. variegatum* females are less visible when fully engorged (unpublished data). This observation may be attributable to the considerable place of *A. variegatum* eggs within the tick body (Fig. 9).

The lobular accessory gland is a distinctive feature of ixodid ticks. This gland results from the maturation of the vestibular vaginal epithelium as the entire female reproductive system develops. Two porose areas are located dorsally on the female basis capituli, below the rostrum (Fig. 1A). During oviposition, these areas are covered by Gené’s organ. The porose areas secrete a substance that is incorporated into the waterproofed wax of the organ of Gené as it everts from the camerostomal fold. In this study, the complete reproductive system of the *A. variegatum* female has not been observed. This could be attributed to the absence of the dissection of a more advanced engorged and mated female (after 15 days of feeding during their rapid engorgement phase, instead of 12 days) or a repleted, newly detached *A. variegatum* female, which would allow full anatomical observation of the mature reproductive system.

#### *Amblyomma variegatum* adults exhibit a blueish hue in the hemolymph

Prior to hemolymph and saliva collection, it is essential to sterilize the tick cuticle to eliminate external bacterial contaminants that could compromise the integrity of internal tissues. In this study, ticks were surface-decontaminated using ultrapure water followed by phosphate-buffered saline (PBS), as previously described [33, 60]. Although some reports suggest that incomplete decontamination does not critically affect internal microbiome profiles, it may introduce bias in the composition of bacterial communities recovered from internal compartments [32]. Recent studies have highlighted that bleach is more effective than ethanol for surface decontamination [11]; however, it may also disrupt internal microbiota. These observations emphasize the need for a standardized, reproducible sterilization protocol.

The collection of hemolymph – the circulatory fluid that fills the tick’s hemocoel – is particularly relevant due to its hemocyte composition, which plays a central role in tick immune responses and interactions with pathogens. Hemolymph components are crucial to understanding bacterial survival and adaptation within the tick environment. As demonstrated in the case of *A. americanum*, the complete depletion of granulocytic hemocytes resulted in the death of the tick following an *Ehrlichia chaffeensis* infection [4]. In this paper, we propose a simple and reproducible method for hemolymph collection by excising the distal tarsus and collecting the exuding fluid. This method is especially useful for partially or fully engorged females. Alternative approaches can be used to maximize hemolymph yield and purity in fed ticks [5]. The hemolymph of insects is characterized by a translucent appearance, ranging from pale amber to green hues, due to the absence of hemoglobin pigment [31]. Interestingly, in adult *A. variegatum*, we observed a distinctive bluish hue during the initial incision, a phenomenon rarely reported in other tick species such as *Haemaphysalis flava*, *Ixodes scapularis*, or *Rhipicephalus microplus* (Fig. 1B) [5, 36, 48, 68]. The underlying mechanism responsible for this phenomenon remains to be elucidated due to the paucity of research focusing on tick hemolymph pigment composition. Additionally, a distinctive odor was noted during dissection, reinforcing the importance of appropriate personal protective equipment, such as a mask, to ensure operator safety.

## Conclusion

The collection of tick organs is crucial for understanding vector-pathogen interactions *in vivo.* In this study, we provide a comprehensive anatomical description of *A. variegatum,* along with practical guidance for isolating key internal structures – most notably the midgut, salivary glands, and ovaries – which play critical roles in vector competence.

The dissection procedures presented here offer a reliable approach to accessing internal tick tissues with a simple, good-quality binocular magnifying glass, and provided that sufficient technical skill is applied to avoid degradation or contamination. Due to the intricate organization and fragility of internal organs, especially in engorged females, meticulous handling is required when preparing specimens for downstream applications.

This foundational work opens the way for future molecular and cellular investigations targeting specific tissues to better understand the physiological responses of ticks to pathogen colonization – at both the biological and transcriptomic levels which seems fundamental because of its major role in transmitting *Ehrlichia ruminantium*.
